# Supplementary figures and images for: Genomic epidemiology of SARS-CoV-2 in Russia reveals recurring cross-border transmission throughout 2020
Source: PLoS One. 2023 May 16;18(5):e0285664. doi: 10.1371/journal.pone.0285664 (PMC10187899; doi:10.1371/journal.pone.0285664)

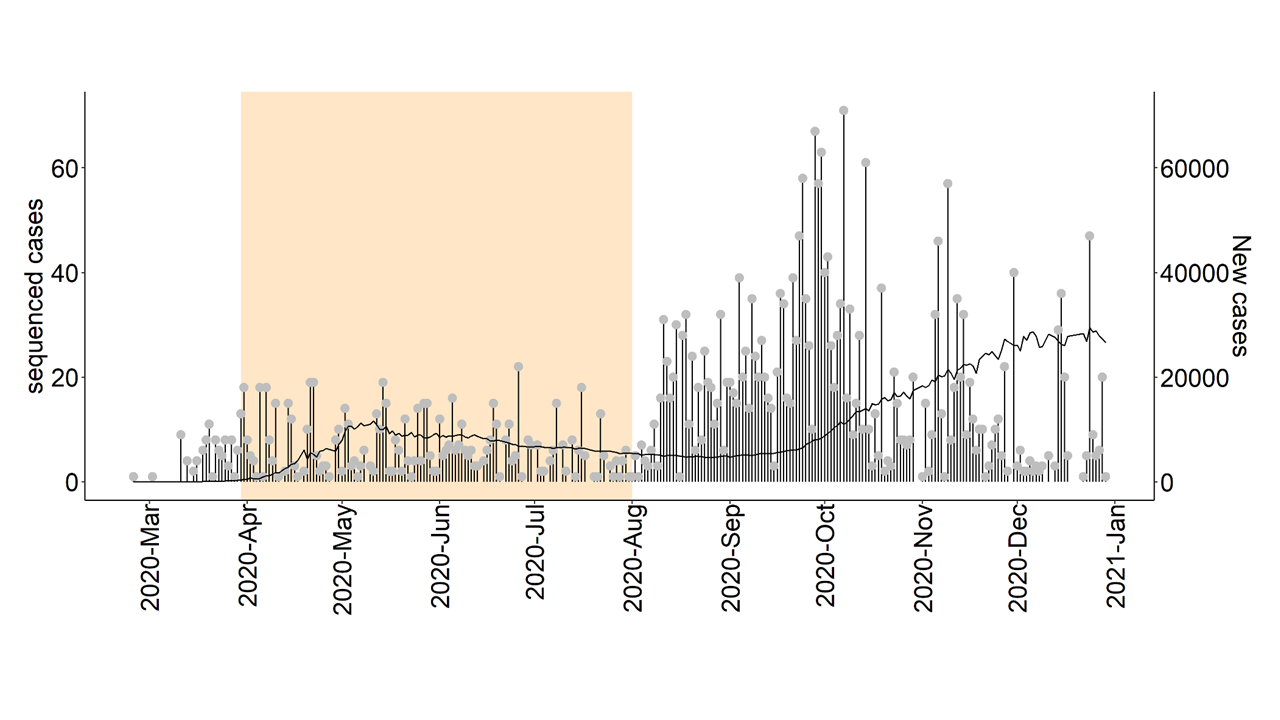

Supplement: S1 Fig — Yellow background indicates the period of the most stringent closure of Russia’s borders. (PNG) [file pone.0285664.s001.png]

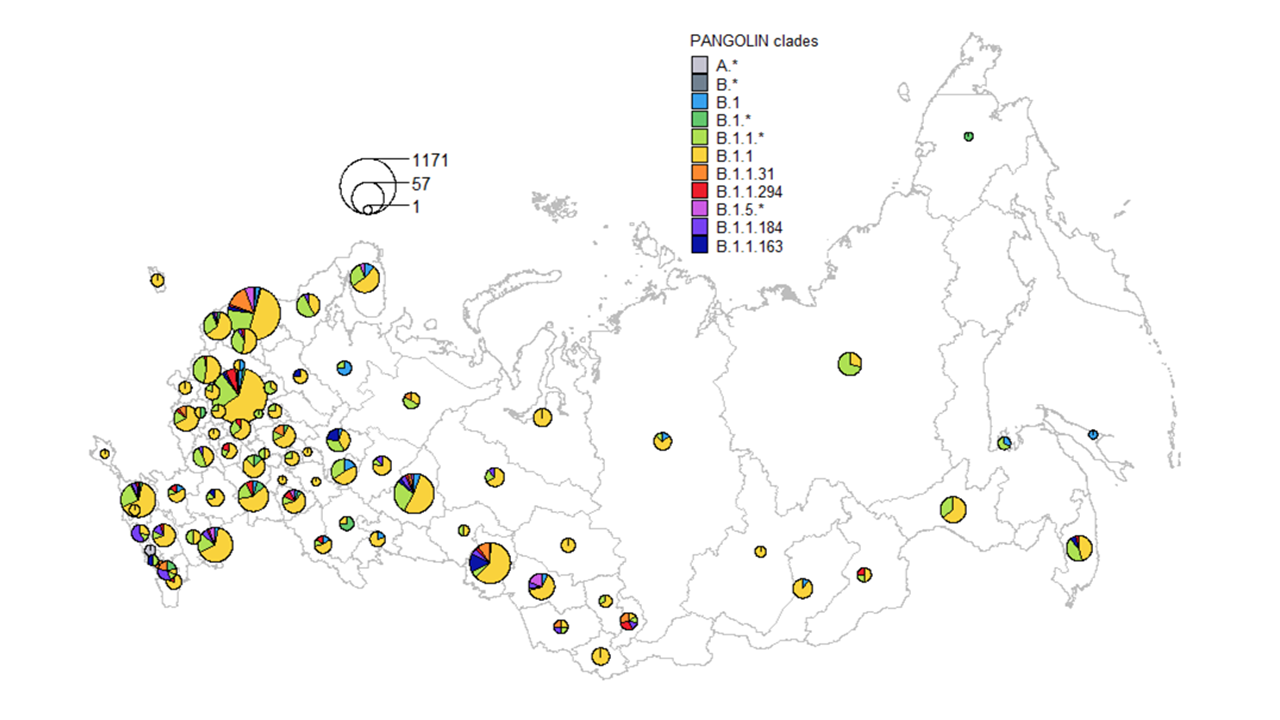

Supplement: S2 Fig — The data presented is for all sequences collected between March-November, 2020; see S1 Fig for a breakdown by period. The circle size is proportional to the number of samples in corresponding regions, categorized by Pango lineages. Moscow is pooled with the surrounding Moscow Region, and Saint Petersburg is pooled with the surrounding Leningrad Region. Asterisks in Pango lineage designations correspond to pooled sets of lineages of that hierarchy level, except those listed in other categories; e.g., B.1.1.* includes B.1.1.7 but not B.1.1 or B.1.1.31. (PNG) [file pone.0285664.s002.png]

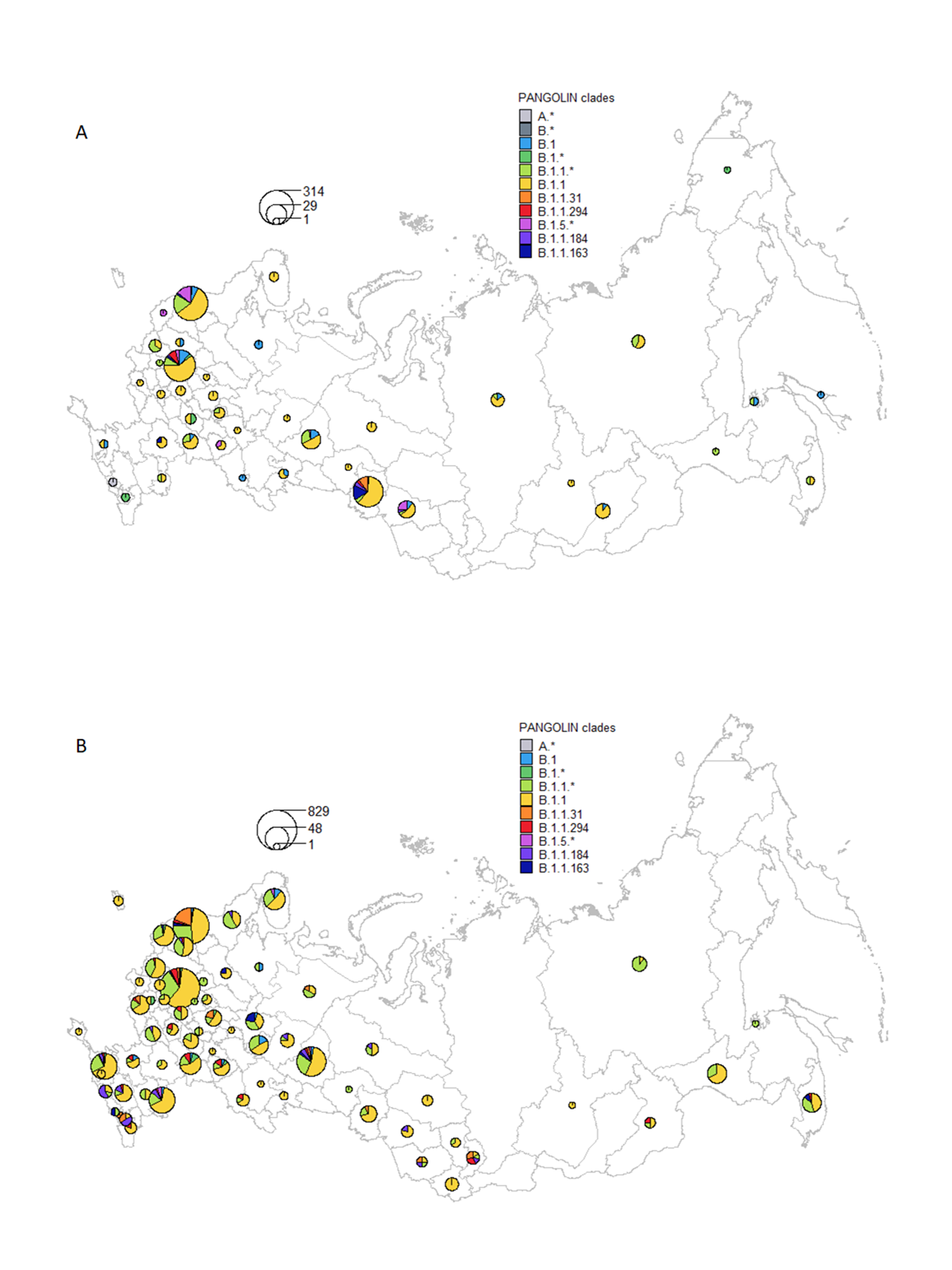

Supplement: S3 Fig — Prevalence of major PANGOLIN lineages [10] in Russia by period: A, March-July; B, August-November. (PNG) [file pone.0285664.s003.png]

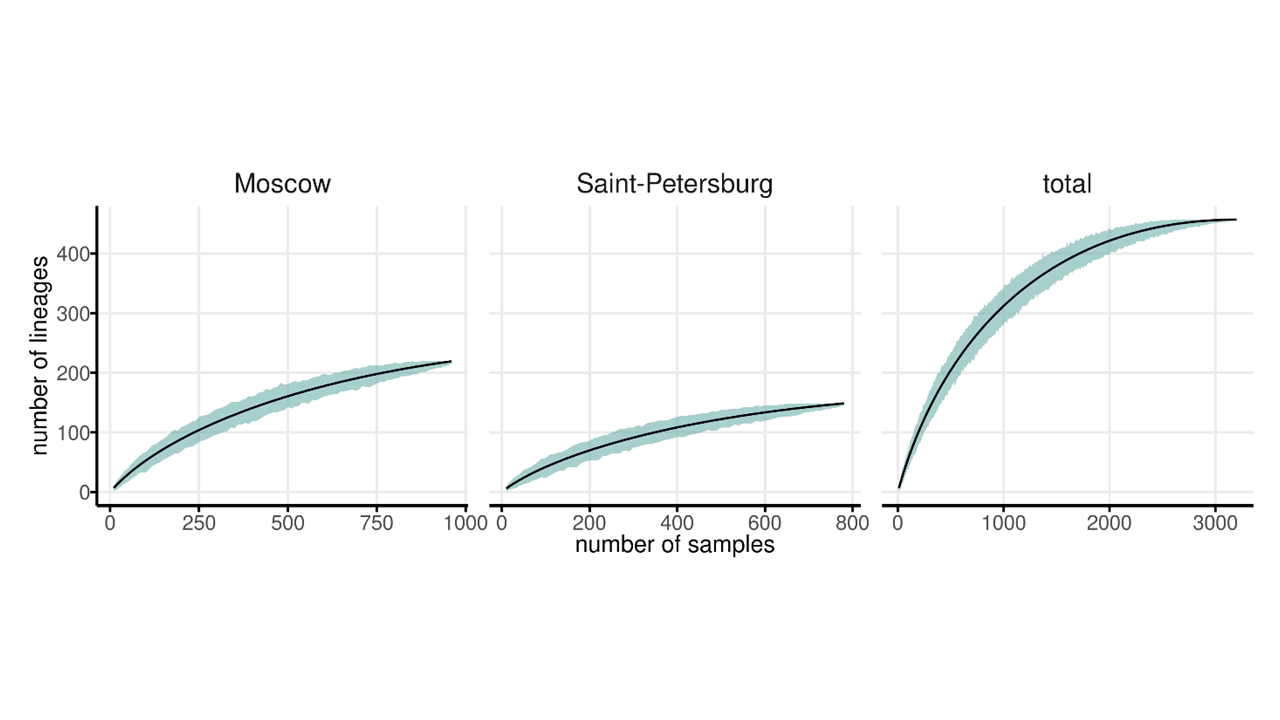

Supplement: S4 Fig — The sequences from Moscow (A), Saint Petersburg (B) and all of Russia (C) were subsampled 10,000 times to the number shown on the horizontal axis, and the number of Russian transmission lineages was inferred. The shaded area shows the range of observed values. (PNG) [file pone.0285664.s004.png]

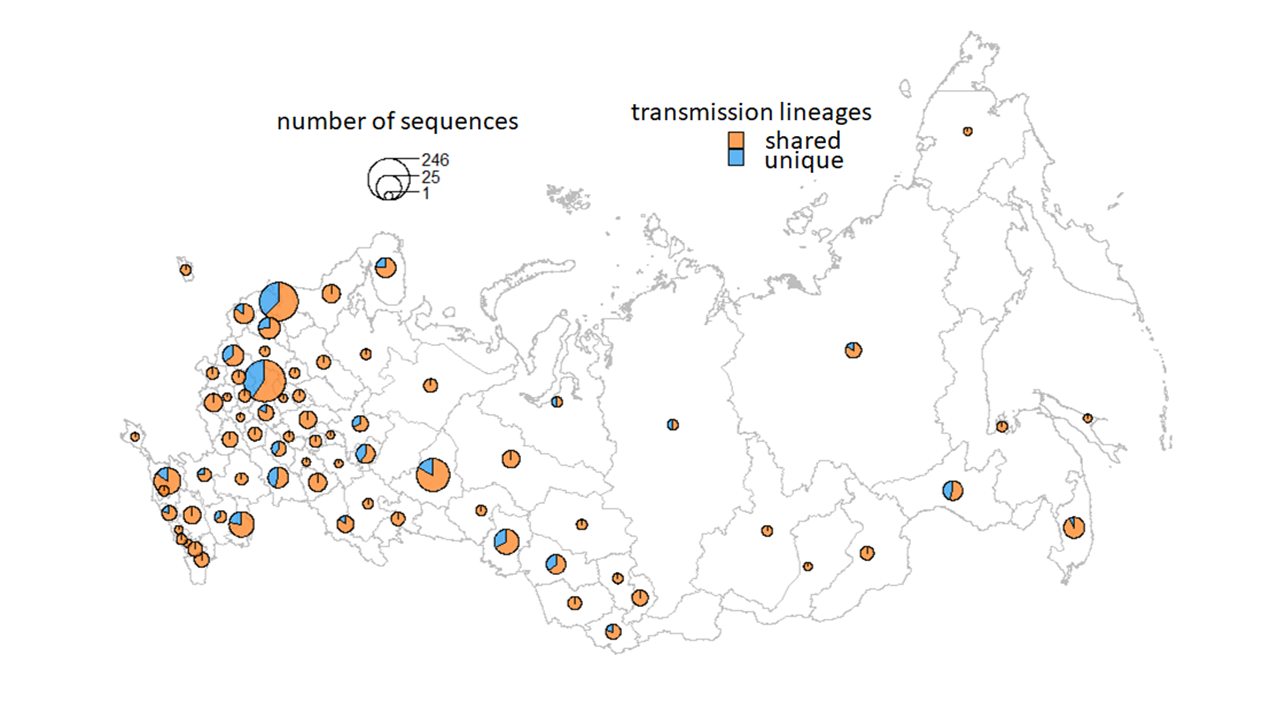

Supplement: S5 Fig — Circle size is proportional to the number of Russian transmission lineages in the corresponding region. (PNG) [file pone.0285664.s005.png]

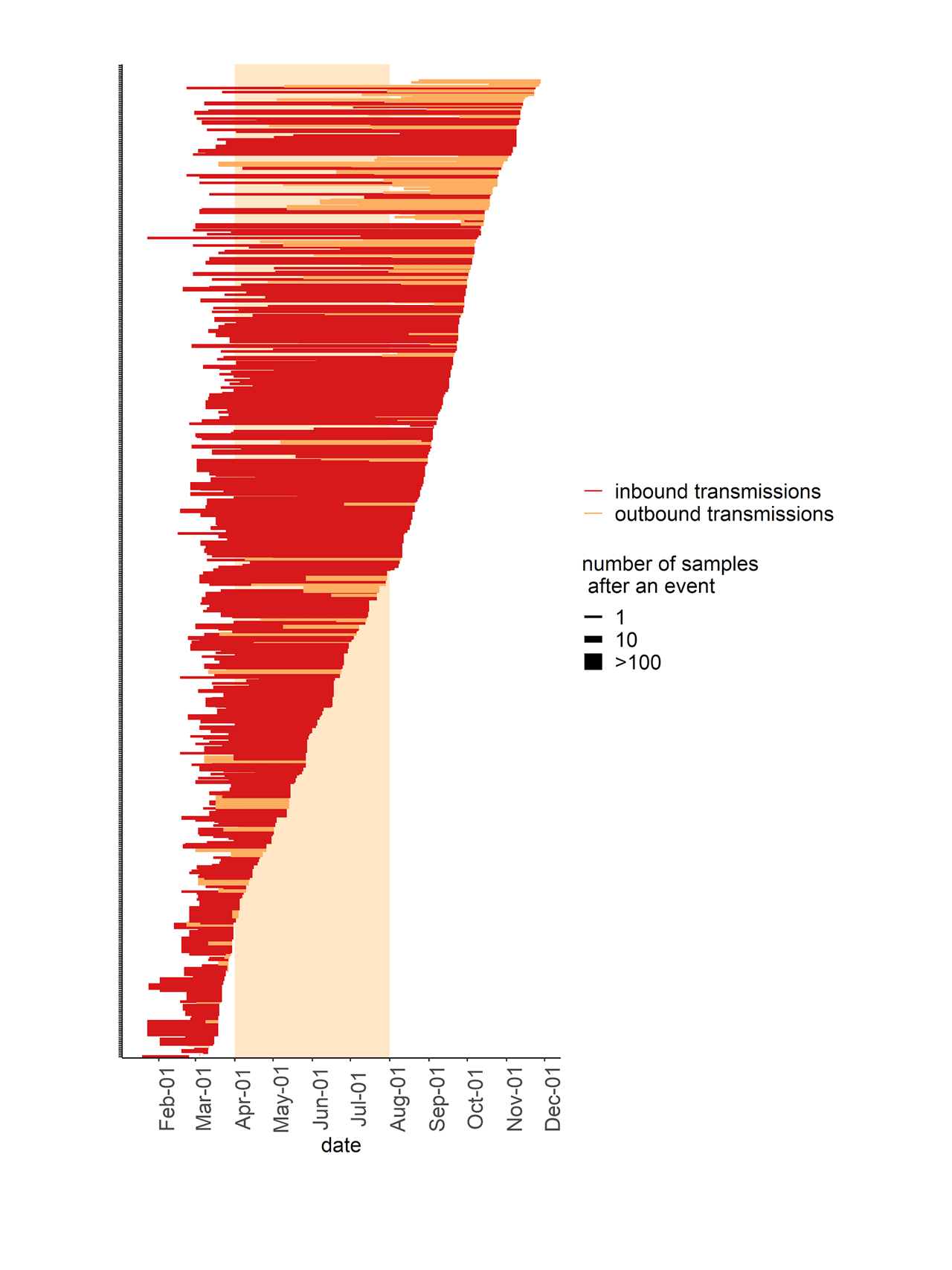

Supplement: S6 Fig — Notations are as in Fig 5. (PNG) [file pone.0285664.s006.png]

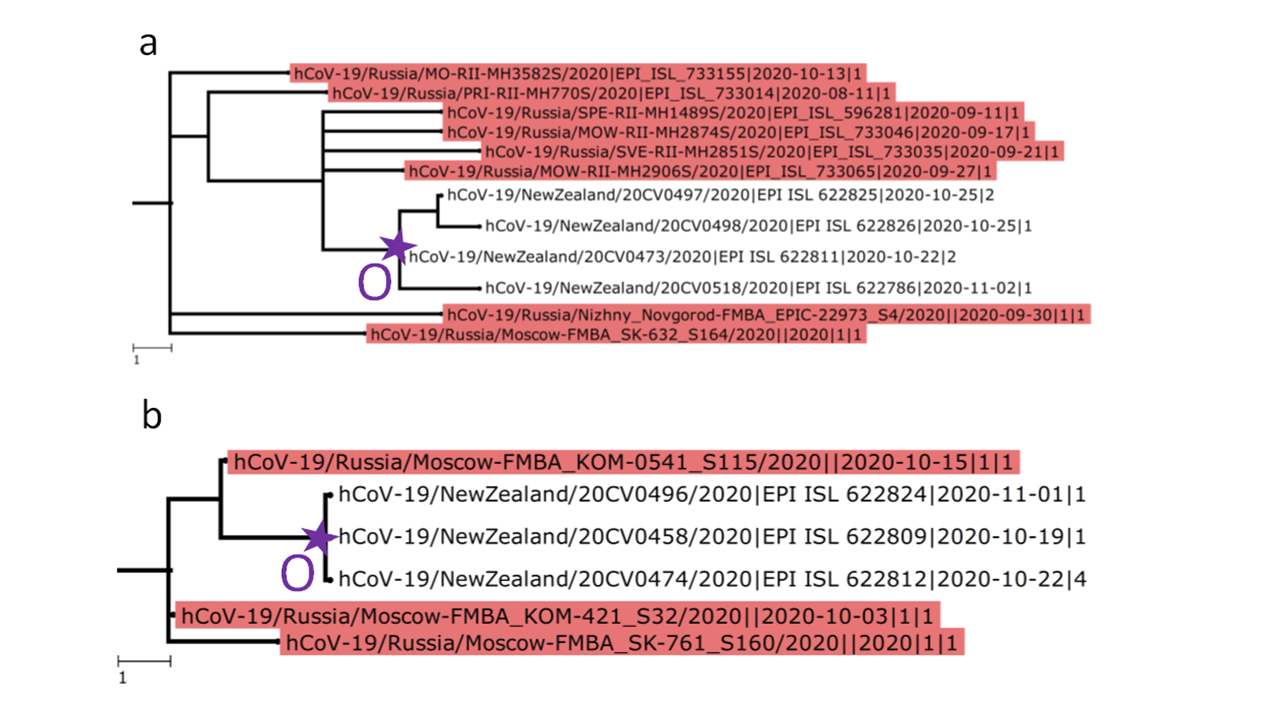

Supplement: S7 Fig — Each tree represents an independent introduction event that occurred in the middle of October, each resulting in 6 sampled sequences. Branch lengths are measured in the number of changes. Samples from Russia are identified with red labels. OBTs are indicated by purple star and “O” letter. The number at the end of the sequence id represents the number of identical sequences (including the one shown) identified in the region on this date. (PNG) [file pone.0285664.s007.png]

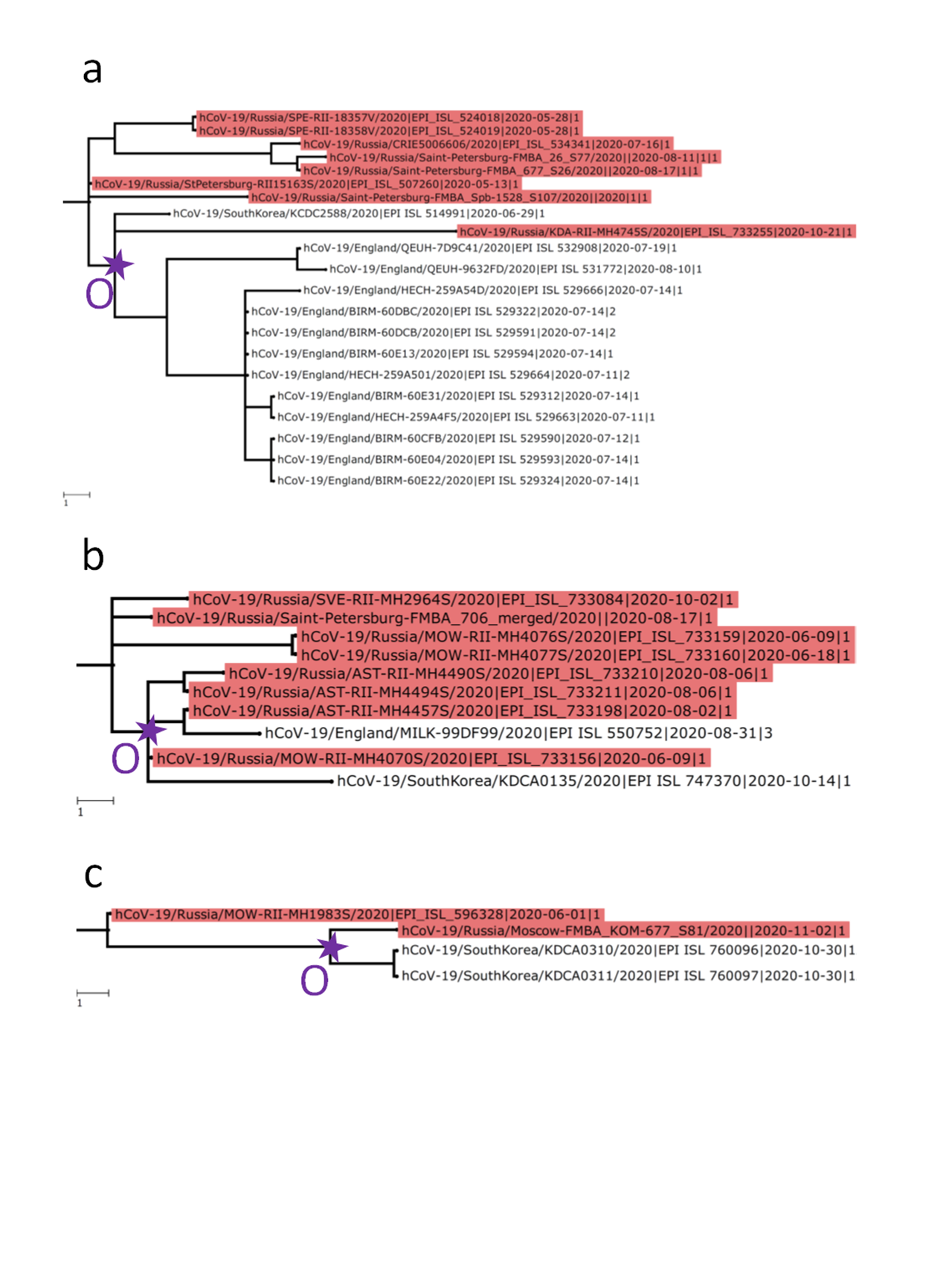

Supplement: S8 Fig — Notation as in S5 Fig. (PNG) [file pone.0285664.s008.png]

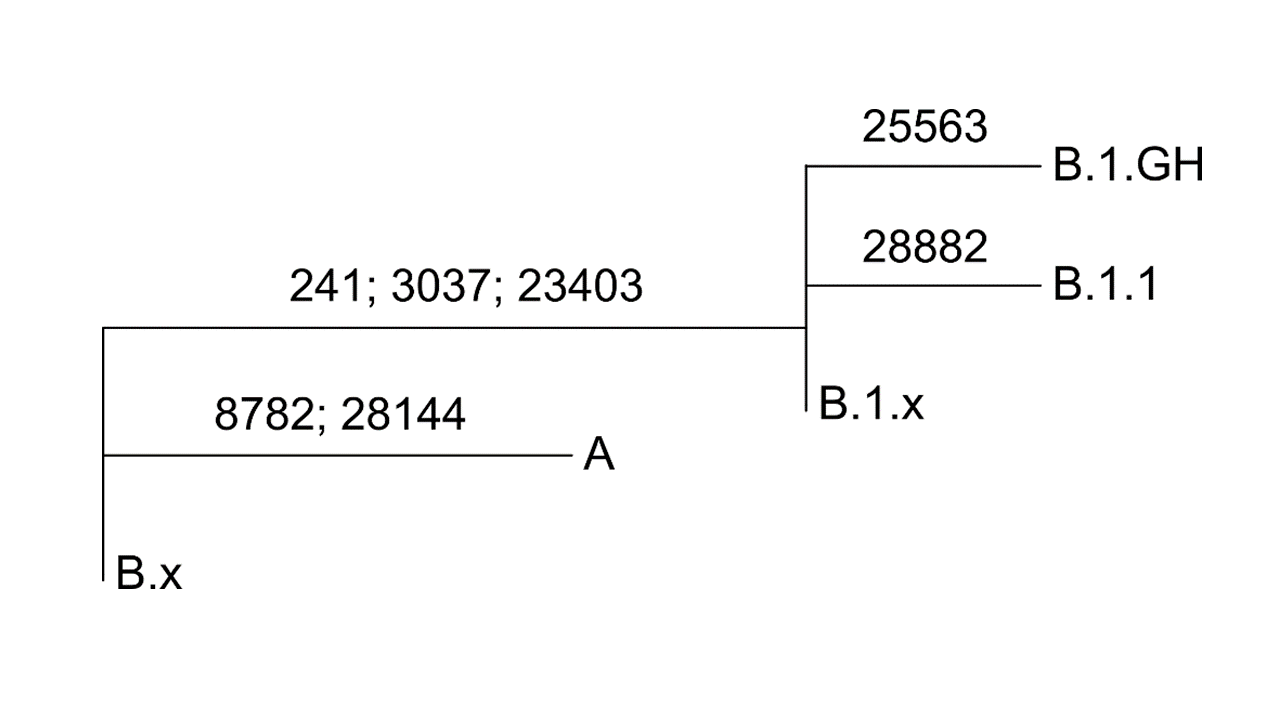

Supplement: S9 Fig — B.x consists of the B lineage and all its descendants except B.1 and lineages descendant from it; similarly, B.1.x includes B.1 and all its descendants but B.1.1 lineage and GH clade (GISAID nomenclature; denoted here as B.1.GH); B.1.1 and B.1.GH clades are analyzed separately. Sites carrying the key mutations defining the specified clades are indicated. (PNG) [file pone.0285664.s009.png]
