# Supplementary material for: Genomic epidemiology of SARS-CoV-2 in Russia reveals recurring cross-border transmission throughout 2020
Source: PLoS One. 2023 May 16;18(5):e0285664. doi: 10.1371/journal.pone.0285664 (PMC10187899; doi:10.1371/journal.pone.0285664)
Supplement: S1 Text — (DOCX) [file pone.0285664.s010.docx]

Table A. Air passenger flow in Russia in 2020 obtained from the Federal Air Transport Agency webpage (<https://favt.gov.ru/dejatelnost-ajeroporty-i-ajerodromy-osnovnie-proizvodstvennie-pokazateli-aeroportov-obyom-perevoz>) 01.09.2022

|  |  |  |  |  |
| --- | --- | --- | --- | --- |
| **year** | **month** | **Passenger service (departed + arrived) (number of passengers)** | | |
|  |  | **International flights** | **Internal flights** | **Total** |
| **2020** | **1** | 4 929 170 | 10 467 763 | 15 396 933 |
| **2020** | **2** | 4 166 359 | 9 804 039 | 13 970 398 |
| **2020** | **3** | 2 505 968 | 9 060 522 | 11 566 490 |
| **2020** | **4** | 30 426 | 1 439 595 | 1 470 021 |
| **2020** | **5** | 48 884 | 1 844 690 | 1 893 574 |
| **2020** | **6** | 69 601 | 5 590 069 | 5 659 670 |
| **2020** | **7** | 110 828 | 13 375 695 | 13 486 523 |
| **2020** | **8** | 912 802 | 17 142 114 | 18 054 916 |
| **2020** | **9** | 1 522 681 | 14 952 022 | 16 474 703 |
| **2020** | **10** | 1 430 033 | 11 237 949 | 12 667 982 |
| **2020** | **11** | 720 836 | 8 287 808 | 9 008 644 |
| **2020** | **12** | 731 440 | 9 181 520 | 9 912 960 |

Table B. Relative rates of cross-border transmission events before and during the travel ban in Russia

| type of events | estimation of dates | number of events before travel restrictions | number of days before travel restrictions | mean number of days between two events before travel restrictions | number of events during travel restrictions | number of days with travel restrictions | mean number of days between two events during travel restrictions | total number of days | total number of events | expected rate of events per day | one-tailed binomial test p-value |
| --- | --- | --- | --- | --- | --- | --- | --- | --- | --- | --- | --- |
| IBT+OBT | first sample | 12 | 19 | 1.6 | 26 | 123 | 4.7 | 142 | 38 | 0.13 | 0.002 |
| IBT+OBT | ML | 40 | 67 | 1.7 | 51 | 123 | 2.4 | 190 | 91 | 0.35 | 0.048 |
| IBT | first sample | 10 | 19 | 1.9 | 22 | 123 | 5.6 | 142 | 32 | 0.13 | 0.0058 |
| IBT | ML | 37 | 67 | 1.8 | 31 | 123 | 4.0 | 190 | 68 | 0.35 | 0.0008 |
| OBT | first sample | 2 | 19 | 9.5 | 4 | 123 | 30.8 | 142 | 6 | 0.13 | 0.18 |
| OBT | ML | 3 | 67 | 22.3 | 20 | 123 | 6.15 | 190 | 23 | 0.35 | 1.0 |
